# Fucoxanthin’s Optimization from *Undaria pinnatifida* Using Conventional Heat Extraction, Bioactivity Assays and In Silico Studies

**DOI:** 10.3390/antiox11071296

**Published:** 2022-06-29

**Authors:** Catarina Lourenço-Lopes, Maria Fraga-Corral, Anton Soria-Lopez, Bernabe Nuñes-Estevez, Marta Barral-Martinez, Aurora Silva, Ningyang Li, Chao Liu, Jesus Simal-Gandara, Miguel A. Prieto

**Affiliations:** 1 Nutrition and Bromatology Group, Analytical and Food Chemistry Department, Faculty of Food Science and Technology, University of Vigo, Ourense Campus, E-32004 Ourense, Spain; c.lopes@uvigo.es (C.L.-L.); mfraga@uvigo.es (M.F.-C.); anton.soria@uvigo.es (A.S.-L.); bernabe.nunez@uvigo.es (B.N.-E.); marta.barral@uvigo.es (M.B.-M.); mass@isep.ipp.pt (A.S.); 2 Centro de Investigação de Montanha (CIMO), Instituto Politécnico de Bragança, Campus de Santa Apolonia, 5300-253 Bragança, Portugal; 3 REQUIMTE/LAQV, Instituto Superior de Engenharia do Porto, Instituto Politécnico do Porto, Rua Dr. António Bernardino de Almeida 431, 4200-072 Porto, Portugal; 4 Key Laboratory of Food Processing Technology and Quality Control in Shandong Province, College of Food Science and Engineering, Shandong Agricultural University, Tai’an 271002, China; ningyangli@126.com; 5 Key Laboratory of Novel Food Resources Processing, Ministry of Agriculture and Rural Affairs, Institute of Agro-Food Science and Technology, Shandong Academy of Agricultural Sciences, Jinan 250000, China; liuchao555@126.com; 6 Key Laboratory of Agro-Products Processing, Technology of Shandong Province, Institute of Agro-Food Science and Technology, Shandong Academy of Agricultural Sciences, Jinan 250000, China

**Keywords:** fucoxanthin, conventional heat extraction, *Undaria pinnatifida*, kinetics, antioxidant activity, neuroprotective activity, antimicrobial activity, in silico studies, docking

## Abstract

Brown macroalgae are a potential source of natural pigments. Among them, *Undaria pinnatifida* is recognized for its high concentration of fucoxanthin (Fx), which is a pigment with a wide range of bioactivities. In this study, three independent parameters were optimized for conventional heat extraction (CHE) to maximize the recovery of Fx from *Undaria pinnatifida*. Optimal conditions (*temperature* = 45 °C, *solvent* = 70%, and *time* = 61 min) extracted 5.1 mg Fx/g dw. Later, the bioactivities of the Fx-rich extracts (antioxidant, antimicrobial, and neuroprotective) were assessed using in vitro and in silico approaches. In vitro assays indicated that Fx has a strong antioxidant capacity and even stronger antimicrobial activity against gram-positive bacteria. This data was supported in silico where Fx established a high binding affinity to DR, a *Staphylococcus aureus* protein, through aa ALA-8, LEU-21, and other alkane interactions. Finally, the in vitro enzymatic inhibition of AChE using Fx, was further supported using docking models that displayed Fx as having a high affinity for aa TYR72 and THR 75; therefore, the Fx extraction behavior explored in this work may reduce the costs associated with energy and solvent consumption. Moreover, this paper demonstrates the efficiency of CHE when recovering high amounts of Fx from *Undaria pinnatifida*. Furthermore, these findings can be applied in different industries.

## 1. Introduction

Algae are an important source of compounds that are beneficial to human health. They have been a part of the Asian diet since ancient times as they have various nutritional properties [1], whereas in western countries, their main applications have been as hydrocolloid agents in the food and pharmaceutical industries [2]. From a nutritional point of view, brown macroalgae, or Phaeophyceae, are interesting due to their high content of macro- and micro-nutrients. They are a natural and reliable source of peptides, amino acids, essential proteins, and minerals, and they have a low lipid content (they contain mainly omega 3 and 6 polyunsaturated fatty acids) [2]. In addition to the previously mentioned nutrients, algae offer a wide range of secondary metabolites that have biological activities, such as vitamins (e.g., vitamin A, E), phenolic compounds (e.g., phlorotannins, phenolic acids), polysaccharides (e.g., fucoidans, carrageenans), or carotenoids (e.g., β-carotene, fucoxanthin) [3]. Regarding Phaeophyceae pigments, especially fucoxanthin (Fx), they have attracted the attention of the academy and industries for its broad biological functional spectra, and for being easily recoverable from natural and sustainable sources such as macro- or microalgae. Among these pigments, is fucoxanthin (Fx). The first time that Fx was isolated was in 1914, and it was extracted from three types of brown algae (*Dictyota*, *Fucus*, and *Laminaria*) in Germany [4]; however, today, *Undaria pinnatifida* algae, also known as Wakame, is a brown algae widely used in Asia, and it is currently distributed throughout the world [1]. Moreover, it is also predominantly used for the extraction of Fx, due to its high content on the lipidic fraction of the pigment [5].

**Figure 1:**
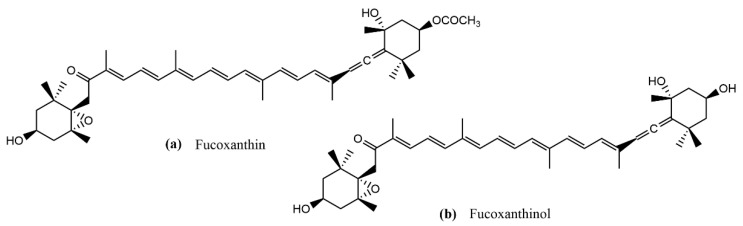
Chemical structure of (**a**) Fx and (**b**) fucoxanthinol.

Fx is considered to be one of the most abundant and characteristic photosynthetic pigments in brown algae, representing approximately 10% of the total carotenoids that exist in nature. Fx has a characteristic orange color, and it belongs to the family of non-provitamin A carotenoids, a class of 40-carbon organic molecules that consist of two groups: carotenes (pure hydrocarbon structures) and xanthophylls (oxygenated derivatives). Fx is a xanthophyll, and its characteristic structure includes an allenic bond, a conjugated carbonyl in the polyene chain, a 5,6-monoepoxide, and hydroxylated and carboxylated residues, which confer antioxidant properties to Fx. Fx absorbs light in the blue–green to yellow–green part of the visible spectrum, peaking at around 510–525 nm according to various estimates, and absorbing light that mainly falls within the 450 to 540 nm range. It behaves as a primary light-harvesting carotenoid that transfers energy to the chlorophyll–protein complex [6]. Since Fx is present in edible algae, it is well known that it undergoes a metabolic transformation after it is ingested by mammals. In mice and rats, the major identified metabolite was fucoxanthinol [7,8]. Fx is transformed into fucoxanthinol due to the actions of digestive enzymes in the gastrointestinal tract. After passing through the tract, it is absorbed in the intestine, and circulated in the bloodstream [9]. The metabolic process mainly consists of deacetylation reactions, thus both molecules, Fx and fucoxanthinol, have similar chemical structures (Figure 1) [10].

Various studies have shown that Fx and fucoxanthinol facilitate important biological and therapeutic activities, such as antioxidant, anticancer, antihypertensive, neuroprotective, anti-inflammatory, anti-diabetic, anti-obesity, and even anti-angiogenic activities [11,12,13,14,15,16,17,18]. Indeed, it has been suggested that fucoxanthinol facilitates even more biological activities than Fx does [10]. The excellent antioxidant properties of Fx that protect against oxidative stress have been repeatedly demonstrated by numerous studies [19], and these properties are derived from its allenic bond and the acetyl functional group that is present in its structure [20]. Fx has been shown to have the ability to inactivate singlet oxygen species and to trap different free radicals, such as DPPH, ABTS, hydrogen peroxide, hydroxyl radicals, superoxide anion, and singlet oxygen [20,21]. The hydroxyl radical scavenging capacities of Fx and fucoxanthinol have been shown to be significantly higher than that of α-tocopherol, although their singlet oxygen inactivation capacities were lower than that of β-carotene. Importantly, Fx acts as an antioxidant under anoxic conditions (i.e., under conditions where there is low dissolved oxygen), whereas other carotenoids, such as β-carotene and lutein, show little or no activity under these extreme conditions [22,23].

The neuroprotective effects of Fx were also studied in in vivo and in vitro models of traumatic brain injuries (TBIs), and the Nrf2-ARE and Nrf2- autophagy pathways were examined as potential promotors of the molecular neuroprotection mechanisms. Results suggest that Fx provides neuroprotection after a TBI, and more specifically, it improves neurobehavioral performance, alleviates brain edema, and decreases the volume of the injury. Furthermore, Fx treatment decreased TBI-induced apoptosis and oxidative stress through the activation of the Nrf2-ARE and Nrf2-autophagy pathways [24].

Taking into account the numerous health benefits offered by the bioactive and nutritional compounds that are present in brown algae, they are ideal substrates for in-depth examinations. Moreover, these traits also enable them to be used as a functional ingredient in innumerable industrial applications, such as in the development of functional foods, pharmaceuticals, and cosmetic products [3,25].

To obtain Fx from algae, various techniques can be used that are both conventional (e.g., maceration, Soxhlet) and innovative (e.g., microwave, ultrasound). In the case of wakame, the use of conventional heat extraction (CHE) (which is similar to maceration but it involves a stirring process) has reported better results during the extraction of Fx than other innovative techniques such as microwave-assisted extraction or pressurized liquid extraction [1]. When using the maceration technique, up to 2.08 mg/g dw was obtained, compared with 0.9 mg/g dw when using microwave-assisted extraction [26]. Moreover, the choice of extractive solvent is a determining parameter in the process, as it is necessary to consider the polarity and solubility, as well as other characteristics, of both the solvent of choice and the target molecule. Additionally, in the food industry, it is important to select solvents that allow for a later extraction to occur in order to avoid toxicity issues. Acetone is a green solvent that is capable of solubilizing Fx, and its use is permitted in the food industry [27]. When using conventional techniques, each extractive process is conditioned by certain variables, such as temperature (*T*), extraction time (*t*), or the solvent dilution (*S*). The different values are represented by these independent variables, and they constitute different conditions that affect the extraction yield; thus it is possible to conduct a mathematical study of the extractive behavior that occurs under the different conditions created by the variables. In this sense, extraction conditions that maximize the yield can be mathematically predicted [28]. There are many mathematical equations that fit experimental data; therefore, the choice of equation depends on the behavior of the experimental data. In most cases, when extracting compounds such as Fx, the kinetic behavior of the compounds follows a first-order process that can be fitted to an equation that uses two parameters. The other variables can be applied to kinetic behavior using other mathematical approaches in a multivariable model. Finally, upon examination of the parametric results, researchers can obtain the optimal conditions that best suit the extraction.

The present work focuses on the optimization of the CHE of Fx from brown algae *U. pinnatifida*, using a kinetic approach and a mathematical equation. This approach allows the behavior of Fx to be defined over time and under different conditions in order to determine the parameters that maximize the extraction yield of Fx. Furthermore, three in vitro bioactivities of Fx—antioxidant, antimicrobial, and neuroprotection—were assessed. The antioxidant response was evaluated by DPPH, ABTS, and Crocin colorimetric assays. Moreover, the antimicrobial and neuroprotective activities were studied using the disk diffusion test and Ellman’s colorimetric method, respectively. The most promising in vitro results were further analyzed by in silico analyses. In silico studies are hugely important in modern science, and they play a significant role in the prediction of the behavior of different molecules. Molecular docking is a useful technique to find new drugs and remedies to fight against diseases and pathogens. This technique consists of predicting the most favorable conformation of a molecule when it binds to a specific target. In this study, molecular docking was conducted by choosing five proteins which were related to the two bioactivities that were experimentally evaluated (antimicrobial and neuroprotective activities), and the binding of Fx to the protein’s active site was analyzed. Additionally, the prediction of ADME (absorption, distribution, metabolism, and excretion) is a helpful tool to predict how compounds will act in the human body, and thus, these characteristics were also studied in Fx by the SwissADME web service.

## 2. Materials and Methods

### 2.1. Sample Collection and Preparation

The studies were carried out using a brown alga from the Phaeophyceae family, specifically *Undaria pinnatifida* (Harvey) Suringar. *U. pinnatifida* was manually harvested from Galician coasts and provided by Algamar (www.algamar.com (accessed on: 28 June 2022). The alga was fresh when it was received, it was cleaned manually by removing the particles adhered to its surface, washed with distilled water, and frozen at −80 °C to better conserve the sample. Then, it was lyophilized (LyoAlfa 10/15 from Telstar, Shanghai, China) and pulverized (~20 mesh). The resulting powder was mixed to guarantee the homogeneity of the samples, it was stored in a freezer (−20 °C), and protected from light, until later analysis.

### 2.2. Conventional Kinetic Heat Extraction

In this study, the kinetic heat extraction of Fx from *U. pinnatifida* was conducted using a conventional extraction method, using a solid–liquid ratio of 30 g/L. Three independent variables were tested: time (*t*, 3 min to 7 days), temperature (*T*, 5 to 65 °C), and concentration of acetone (*S*, 50 to 100%). Different aqueous dilutions of acetone were prepared. The solvent proportions used were 50, 60, 70, 80, 90, and 100 % of acetone. For each *S* proportion, the following combinations of *T* and *t* were applied: 5 °C (30, 120, 480, 1200, 2640, 4200, 5700, 9680 min), 25 °C (15, 30, 120, 480, 1200, 1680, 2640 min), 45 °C (3, 5, 15, 60, 210, 1200, 1680, 2640 min), and 65 °C (3, 5, 15, 30, 60, 120, 210, 480, 1200, 1680 min). In this design, a total of 198 experimental points were generated. Six amber bottles, with the respective acetone concentrations, were placed in 4 water baths at the previously mentioned temperatures. Once the solvent reached the desired temperature, the alga powder was added to the bottles at time 0 and stirred at 800 rpm, using Thermo Scientific™ Cimarec™ i Micro Stirrers. Samples were collected from each bottle at the predetermined collection times and centrifuged for 7 min at 8400 rpm. The supernatant was then filtered, with Ø 0.22 µm nylon syringe filters, into vials. Dry weight (dw) was calculated for each of the experimental points, and then expressed in mg of extract (E) per g of the algae sample’s dry weight (AS dw).

### 2.3. Fucoxanthin Detection and Quantification through HPLC

The HPLC method used to quantify the Fx present in each sample was performed with Waters HPLC equipment (including a Waters 600 controller, Waters 600 pump, Waters 2996 photodiode array detector (1.2 nm optical resolution), Waters 717 plus autosampler, and a Waters AF in-line degasser). For the stationary phase, the analytical separations were performed using a Waters Nova-Pak C18 column (150 × 3.9 mm, WAT 088344). The column was stabilized at 25 °C. The mobile phases used were as follows: eluent A was a solution of 5 mM ammonium acetate in milli-Q water, eluent B was a solution of 5 mM ammonium acetate in methanol, and eluent C was pure ethyl acetate. The organic solvents used to prepare the mobile phases were HPLC grade. Ammonium acetate solutions (5 mM) were prepared as follows: 385.4 mg of ammonium acetate were dissolved in 1000 mL of the corresponding solvent (milli-Q water or methanol) using a magnetic stirrer.

The flow rate was set at 0.5 mL/min and the injection volume was 50 µL. The elution gradient established was 5% A and 95% B up to 8 min, 50% B and 50% C up to 20 min, 50% A and 50% B up to 35 min, and 30% A and 70% B until the end of the 40 min run. The detection of Fx was conducted by a photodiode array detector (DAD) with absorbances between 450 nm and 700 nm. The quantification of the Fx content analyzed by HPLC-DAD was expressed as µg Fx/g AS dw, (Table A1). The Fx standard was purchased from Sigma.

### 2.4. Statistical Analysis, Mathematical Modeling and Graphical Representation

The different values that are represented by the independent variables (*T*, *S*, and *t*) constitute the several conditions that can affect the extraction yield; therefore, it is possible to conduct a mathematical study concerning Fx extraction behavior using different variable conditions. In this sense, the extraction conditions that maximize the yield can be mathematically predicted [28]. Thus, a mathematical model that describes all the variables studied is needed to empirically find the optimal conditions for the extraction of Fx. The behavior of the experimental data shows, in all cases, a first-order structure that can be fitted with two parameters Equation (1):

(1) $$R=k\;(1-e^{-rt})$$

where *R* (µg Fx/g AS dw) is the dependent variable, *k* (µg/g) is the maximum amount of Fx that can be extracted at a determined temperature and concentration of acetone, and *r* (min^−1^) is the rate constant that provides information about the extraction rate. Regarding *k*, the amount of Fx extracted is higher when the value *k* increases; however, in the case of *r*, when this value increases, a shorter extraction time is required to reach the maximum amount of Fx.

To calculate the extraction time (*t*) at a certain temperature and solvent concentration, Equation (2) was used, as follows:

(2) $$t\;=\frac{{\mathrm{Ln}\;(2}^{n})}{r}$$

where *r* is the kinetic constant, and *n* is the number of semi-extraction periods that have elapsed. In this study, ten semi-extraction periods were used, which refers to the time needed to extract 99.902% of Fx. With this equation, when using higher extraction rates, lower *t* can be observed.

All adjustment procedures, coefficient estimates, and statistical calculations were performed using a Microsoft Excel spreadsheet. The adaptation and statistical analysis of the experimental results, in accordance with the proposed equations, were conducted in four phases:

- Determination of the coefficients: the parametric estimates were obtained by minimizing the sum of the quadratic differences between the experimental values and those predicted by the model, using the non-linear method of least squares (quasi-Newton) provided by the macro Solver in Microsoft Excel 2003 [29], which allows for the rapid analysis of a hypothesis and its consequences [30].
- Significance of the coefficients: the determination of the confidence intervals of the parameters was carried out using ‘*SolverAid*’ [31]. The model was simplified, discarding the terms that were not statistically significant for the *p*-value (*p* > 0.05).
- Model consistency: Fisher’s F test (α = 0.05) was used to determine the adequacy of the models built for the data obtained [32].
- Other statistical evaluation criteria: to re-verify the uniformity of the model, the following criteria were applied: (i) the macro ‘*SolverStat*’ was used [33] to evaluate the prediction uncertainties of the parameters and models; (ii) the *R*^2^ was interpreted as the proportion of versatility of each dependent variable that was described in the model; (iii) the adjusted coefficient of determination (*R*^2^*adj*) corrected *R*^2^, taking into account the number of variables used in the model.

The graphical representation of the results obtained was conducted using Sigma Plot 14.0 program.

### 2.5. Evaluation of the Biological Properties of the Optimized Extract

Optimized extracts, rich in Fx, were used to evaluate different bioactivities, such as antioxidant, antimicrobial, and neuroprotective activities, through several assays that are described below. 

#### 2.5.1. Antioxidant Activity

##### DPPH Radical-Scavenging Activity

The antioxidant capacity was determined using the methodology proposed by [34], with some modifications. A stock methanolic solution (76 mM) of the DPPH radical was prepared. Then, the stock reagent was diluted with methanol at a ratio of 1:50 to obtain an absorbance between 1.2–1.3 units that measured at 515 nm. The reaction was conducted in 96-well microplates, and 40 µL of each extract (diluted in 7 different concentrations of the respective solvent) were mixed with 200 µL of the DPPH reagent. The reaction mixture was incubated at room temperature in the dark for 60 min. Finally, the absorbance at 515 nm was measured using a Synergy™ HTX microplate reader (Agilent, Santa Clara, CA, USA). The assay was produced in triplicate.

##### ABTS Radical-Scavenging Activity

This methodology is based on the ability of the antioxidants present in the alga extract to sequester the ABTS^•+^ radical, in comparison to the sequestering ability of the Trolox compound, an analog of water-soluble vitamin E [35]. The technique was carried out following a methodology proposed in the literature [36]. Moreover, the ABTS^•+^ radical was generated from the interaction between 21.95 mg of ABTS dissolved in 10 mL (4 mM) of ultrapure water and 4,06 mg of potassium persulfate (K_2_S_2_O_8_) (1.5 mM) dissolved in 10 mL of ultrapure water. The mixture was incubated in the dark at room temperature for 16 h. Then, the stock solution was diluted with ethanol at a 1:10 ratio to obtain an absorbance between 1.3–1.4 units that measured at 734 nm. The reaction was initiated after 190 µL of the previously prepared ABTS^•+^ radical solution and 10 µL of each extract (diluted in 7 different concentrations of the respective solvent) was added to the mixture. After a 6 min incubation at room temperature, the absorbance at 734 nm was measured using a Synergy™ HTX microplate reader. The assay was produced in triplicate.

##### Crocin Bleaching Assay (CBA)

This method was first proposed by [37] and it uses crocin as an oxidizable substrate and AAPH (2,2′-azobis-2-amidinopropane: RN = NR) as a source of radicals for which crocin and the antioxidant compete. Moreover the presence of AAPH reduces the discoloration rate of the crocin. For this, a stock solution of 5 mg of crocin in 25 mL of ultrapure water at 40 °C was prepared, as well as a solution of 75 mg of AAPH in 5 mL of ultrapure water at 40 °C. After both substances were completely dissolved, the solutions were mixed together; with this method, an absorbance of ~1,4 was obtained. Moreover, 250 µL of this solution were used immediately in each well of the microplate, which was prepared in advance, to start the reaction. The microplate had 50 µL of 7 concentrations of the antioxidant that was being studied, as well as a blank. The absorbance was read at 0 min and the microplate was left to incubate in an agitated state. It was protected from light at 37 °C, and was read in 30 min intervals until complete discoloration of the blank, at 450 nm. The assay was produced in triplicate.

#### 2.5.2. Antimicrobial Activity

The dry extracts of the samples were dissolved in water (10 mg/mL), and for the evaluation of antimicrobial activity, the procedure described by [38] was followed. The determination of antimicrobial activity was tested via culturing in a Petri dish and measuring the inhibition halos produced by the addition of the extracts [39,40]. The bacteria used are some of the most common microorganisms [41]. The extracts’ activity was studied against three gram-negative bacteria, *Escherichia coli*, *Salmonella enteritidis*, and *Pseudomonas aeruginosa*, and three gram-positive bacteria, *Bacillus cereus*, *Staphylococcus epidermidis*, and *Staphylococcus aureus*.

The bacteria were first inoculated in a tube with 10 mL of MHB, and they were left to grow between 12 and 24 h, at 37 °C. The evaluation of the number of colonies was carried out using UV spectrometry at 600 nm, and it was set between 1 and 2 × 108 colony-forming units (CFUs), with adjustments made to the absorbance between 0.09 and 0.110 of the Mcfarland scale, following the standardized method [42]. In a Petri dish, 100 µL of the previously cultivated inoculum was deposited and streaked in 4 quadrants. Subsequently, 15 µL of dimethyl sulfoxide (DMSO) was added as negative control and 15 µL of 40% lactic acid was added as a positive control. In each of the remaining quadrants, another 15 µL of the extract was added (20 mg/mL in DMSO). The incubation lasted 24 h at 37 °C. The inhibition halos were measured with a pachymeter in millimeters. The assay was produced in triplicate.

#### 2.5.3. Neuroprotective Activity

A previously developed colorimetric method was used [43]. It consists of detecting the inhibition of the activity of AChE and BuChE, which occurs due to the increase in yellow coloration as a result of the production of thiocholine. Both enzymes are involved in the mechanisms of Alzheimer’s disease, and AChE inhibition has been recognized as a possible route for the symptomatic treatment of this disease [44]. To produce this assay, 3 buffers were used: buffer A (50 mM Tris-HCl, pH 8), buffer B (50 mM Tris-HCl, pH 8 0.1% BSA), and buffer C (50 mM Tris—HCl, pH 8, 0.1 M NaCl and 0.02 M MgCl2). Acetylcholine and donepezil were used as controls. The inhibitory capacity of the extracts was tested at concentrations of 1 and 2 mg/mL. The mixture of buffers, reagents, and the sample was distributed in 100 µL volumes (50 µL buffer, 25 µL enzyme, and 25 µL sample) in a 96-well microplate, for which a control and a reagent blank had also been prepared. The optical density was determined by spectrophotometry at 412 nm. Optical density readings were conducted every minute from 0 to 20 min, and the mean increase in absorbance per min was calculated, which indicates the reaction rate of the process. The assay was produced in triplicate for each buffer, and the mean values were calculated between the replicas and each buffer used. The inhibitory activity values were calculated in accordance with Equation (3):

(3) $$I\;(\%)=\frac{(AbsC-AbsCB)-\;(AbsM-\mathrm{AbsCB})}{(AbsC-\mathrm{AbsCB})}\times 100$$

where AbsC represents the mean absorbance per minute of the control, AbsCB represents the mean absorbance per minute of the control blank, and AbsM represents the mean absorbance per minute of the sample.

### 2.6. In Silico Studies: Molecular Docking and Pharmacokinetic Study

#### 2.6.1. Molecular Docking

##### Fucoxanthin (Ligand) Preparation

The two-dimensional structure of Fx was downloaded from PubChem in a SMILES (Simplified Molecular Input Line Entry) format. Next, it was converted to a three-dimensional structure using ACD/ChemSketch version C15E41, and saved in a “.mol” format. The three-dimensional models were optimized using VEGA ZZ 3.2.1 software and they were saved in “.pdb” formats. After the model optimization, the three-dimensional optimized Fx model was introduced as a ligand using AutoDookTools 1.5.6 software, and converted to “.pdbqt” format. At this point, the Fx was prepared for molecular docking.

##### Protein Preparation

The proteins selected for the docking were: acetylcholinesterase (PDB:4EY7, AChE), butyrylcholinesterase (PDB: 1P0P, BuChE), beta-ketoacyl-(acyl carrier protein) synthase I (beta-ketoacyl-ACP synthase I) (PDB:1FJ4, KS), ADN gyrase (PDB:2XCS, GY), and dihydrofolate reductase (PDB:3SRW, DR). Before the protein preparation, a Ramachandran plot was produced in order to examine the integrity of the torsional angles of the proteins. To prepare the proteins for docking, the structures were first downloaded from the Protein Data Bank (PDB) in a “.pdb” format. Then, the ligands that were complexed with the proteins were separated in Wordpad. The ligands that were separated from the proteins followed the same steps as Fx, with regard to its optimization using the AutoDookTools 1.5.6 software. The proteins without the complexed ligands were also prepared for docking using AutoDookTool 1.5.6 software, wherein polar hydrogens were added to the protein structure, Gasteiger charges were computed, and AD4 atoms were assigned. At this point, all the components for molecular docking were ready.

##### Molecular Docking

The inhibitory effect of Fx was calculated by measuring the binding affinity at the time when Fx was optimally joined to the active site of the selected proteins. To indicate to the software that will carry out the molecular docking (Autodook Vina) where the active sites of the proteins are, PyMOL software was used [45]. The coordinates of every active site were given by the coordinates of the central atoms of the ligands that were complexed with the proteins. At the moment when the coordinates were known, a virtual box of 25 Å was created on each site that had the coordinates of the central atom of the complexed ligand at the center. This virtual box only delimitates the molecular docking simulation inside of the virtual box (active site). Then, molecular docking was carried out using Autodook Vina software. The results are shown as kcal/mol and as a “.pdbqt” field, with the coordinates of Fx in its optimal binding conformation. To validate the methods, the RMSD was calculated between the real position of the complexed ligand of AChE and the simulated position.

##### Results Analysis and Visualization

With the binding affinity provided by Autodok Vina, an estimation of the inhibition constant of the proteins and the Fx was calculated with Equation (4). Moreover, the BIOBIA Discovery Studio Visualizer v21.1.0.20298 software was used to visualize the “.pdbqt” file that was generated during the docking.

(4) $$Ki=Kd=\exp\left(\frac{\Delta G}{RT}\right)$$

#### 2.6.2. Pharmacokinetics Study

For the in silico prediction of the comportment of Fx in the organism, in terms of its absorption and toxicology, the online software SwissADME was used [46]. The 2D structure of Fx was uploaded to the SwissADME server, and the results were downloaded as a table and in a ‘boiled egg’ format. This study produced significant results with regard to the predictions made, which were as follows: gastrointestinal absorption, blood–brain barrier permeation, the production of p-glycoprotein substrates and cytochrome P450 inhibitors, and skin permeation.

## 3. Results and Discussion

### 3.1. HPLC Results

In this study, the kinetic extraction of Fx was carried out by subjecting *U. pinnatifida* to conventional extraction in a solid–liquid ratio of 30 g/L. Three independent variables were evaluated (*t*, 3 min to 7 days; *T*, 5 to 65°C; *S*, 50 to 100% (*v*/*v*)), as observed in Table A1, thus enabling the optimal values for obtaining Fx to be determined. The Fx content was analyzed using HPLC-DAD and expressed in µg Fx/g of AS dw. The results obtained are presented in Table A1.

Overall, these results show that Fx can be extracted even at low temperatures and with short extraction times. Nevertheless, for better results, temperatures around 45 °C, and times greater than 1200 min are recommended. When analyzing these results, we can see that the Fx molecule presents a more robust stability compared with how it is described in the literature, as it resists quite high temperatures (such as 65 °C) for several hours, without suffering degradation. In addition, different extraction conditions can be proposed, depending on the variables of interest in the industrial process. For example, a factory could use longer extraction times at lower temperatures, or vice versa; the extraction process can be adapted to ensure that the most profit is made, even if the final yield is lower.

Following the objective criteria, we can observe in Table A1 that the best results were obtained with a temperature of 45 °C, with times between 210 and 1680 min. Additionally, it is evident through a preliminary analysis, that higher yields were obtained at a solvent concentration of acetone that was between 60% and 80%. Under these conditions, the extraction yield of Fx reached 5.57 mg Fx/g AS dw, with 70% acetone, for 1200 min, at 45 °C.

### 3.2. Analysis of the Kinetic Parameters and a Search for Optimal Conditions

**Figure 2:**
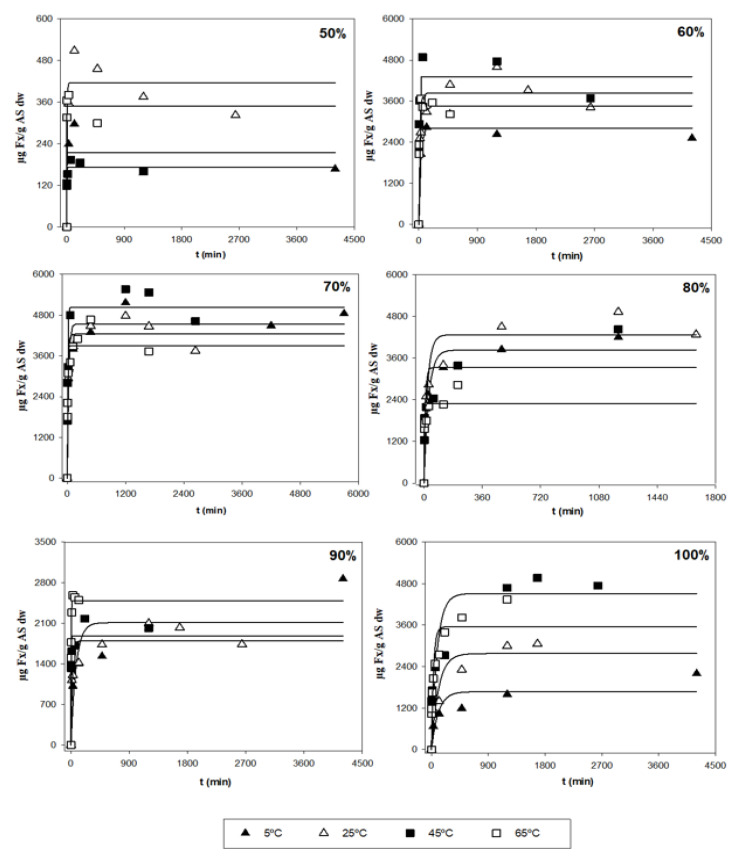
Representation of the experimental data illustrating the amount of Fx presented in one gram of the alga sample (*Y*_1_. symbols) and model-predicted data (lines) as a function of the acetone concentration (*S*).

After the first evaluation of the obtained data, a more thorough analysis was performed in order to determine the optimal extraction conditions for the three responses (*Y*_1_, *Y*_2_ and *Y*_3_), based on the kinetic parameters and their statistical analyses. Table 1 shows the parametric values, *k* and *r*, with 95% confidence intervals for the three different responses (*Y*) at different *T* and *S*, as determined by the tool, Solver. Values that were not statistically significant (ns) were not considered when finding the optimal conditions. According to the table, a high diversity in values can be observed in terms of the different types of responses. *Y*_1_ is related to the amount of Fx present in one gram of the alga sample (µg Fx/g AS), in which the ranges of *k* varied between 415 and 5029 µg Fx/g AS, and between 0.001 and 0.304 min^−1^ for the kinetic parameter *r* (Figure 2). The highest values of parameter *k* and *r* were 5029 µg/g and 0.304 min^−1^, respectively.

Regarding *Y*_2_, this response is related to the yield, which means the amount of extract per gram of alga sample (mg E/g AS). The ranges of *k* fluctuated between 380 and 487 mg E/g AS, whereas the *r* parameter varied between 0.005 and 2.486 min^−1^. The highest values of *k* and *r* were 487 mg E/g AS and 2.486 min^−1^, respectively. Finally, *Y*_3_ indicates the purity (*Y*_1_/*Y*_2_) of the extract, which means the amount of Fx per gram of extract (mg Fx/g E). The values of parameter *k* varied between 6.4 and 109.2 mg Fx/g E, whereas the values of *r* oscillated between 0.012 and 0.065 min^−1^, which were the highest values of *k* and *r* at 109.2 mg Fx/g E and 0.065 min^−1^, respectively.

Table 2 shows the MAE, RMSE, and RMSE-MAE values for the three responses (*Y*) at different *T* and *S*. These error measures allow the ability of the first-order kinetic equation (1) to be evaluated in terms of how well it fits the experimental data. With this in mind, MAE, and RMSE were used to evaluate the grade of error between empirical and experimental values. It can be observed that, generally, the MAE and RMSE values were below 500 and 250 for the RMSE-MAE values at any *T* and *S* for *Y*_1_. Hence, the magnitude of the fitting errors was not significant. In the case of *Y*_2_ and *Y*_3_, in most cases, the magnitude of errors was low; therefore, the observed values were close to the model-predicted values. According to these results, it can be stated that the precision in which the model predicts the response is quite good; thus, mathematical equation (1) can suitably fit the experimental data with very good reliability.

Table 3 shows the trend of the parameters *k* and *r* in conjunction with *T* and *S* (i.e., it illustrates how to vary the parametric values with changing *T* and constant *S*, or with changing *S* when *T* is constant). In the case of *Y*_1_, the *k* parameter did not present significant variations when using different *T* with constant *S*; therefore, *k* is independent of *T*. Conversely, the *r* parameter depends on *T*, because the higher the *T*, the higher the extraction rate for any *S*. In fact, generally, the highest value of *r* was found when using *T* = 65 °C (Table 1). On the other hand, when *T* remains unchanged, *k*_max_ was reached when using medium acetone concentrations. In fact, the most significant *k* values were found when using *S* = 70% at any temperature, as their values were above 3900 µg/g (Table 1); therefore, *k* depends on *S*. Regarding the other parameter, *r* presents variations when *S* changes; more specifically, when *S* increases, the value of *r* decreases. In fact, the lowest values were found when using *S* = 100% (Table 1); therefore, it can be stated that this kinetic parameter (*r*) depends on *S*. Regarding *Y*_2_, when using the same *S*, the values of *k* did not undergo substantial changes when varying *T*; therefore, *k* is independent of *T*. However, the values of *r* depend on *T*. They increase with *T*, reaching, generally, their maximum values when applying temperatures above 45 °C. When attempting to preserve the same *T*, it can be stated that the parametric value *k* decreases when the amount of used solvent is increased. The most significant *k* values were found when using low-medium acetone concentrations (<80%) for any temperature in which their values varied between 380 and 487 mg E/g AS. In addition, it can be observed that the values of *r* show the same abovementioned trend, in which higher values are usually found when using lower acetone concentrations (Table 1); therefore, *r* depends on *S*. Finally, in the case of *Y*_3_, when *S* is constant, *k* remains constant for different *T*. Hence, *r* depends on *T*. When *T* is constant, it can be observed that the values of *k* increase with *S*, reaching the highest values when *S* = 100%. In the case of *r*, for both parameters, the values were not considered because many values were not statistically significant (Table 1).

On an industrial scale, desirable conditions are based on finding a process in which the maximum Fx amounts (*k*) are obtained with the highest possible extraction rates (*r*). With this in mind, it can be stated that the best conditions are those ranges of *T* and *S* in which the values of *k* and *r* are the highest possible. According to Table 1, using a temperature of 45 °C with 70% of acetone were ideal conditions for extracting the highest amount of Fx (*Y*_1_) in the shortest time possible; in this instance, the values of the kinetic parameters were 5029 µg Fx/g S and 0.113 min^−1^. In addition, the *R*^2^ for these conditions was quite good (above 0.950), and the RMSE-MAE values were not very high (Table 2). Regarding *Y*_2_, it can be stated that using a *T* = 45 °C with 50% and 60% of acetone were the best conditions for obtaining the highest possible yields. In fact, the values of *k* and *r* were 468 and 463 mg E/g AS, and 2.486 and 2.129 min^−1^, respectively. Furthermore, the *R*^2^ for both parameters was above 0.970, and the RMSE-MAE values were below 2.5 (Table 1). Finally, in the case of purity (*Y*_3_), the conditions required to obtain the purest Fx extract were 25 °C and 65 °C with 100% of acetone, and the values of *k* and *r* were 70.1 and 105.4 mg Fx/g AS, and 0.049 and 0.065 min^−1^, respectively; however, their *R*^2^ values were quite low (below 0.750) and their RMSE-MAE values were very high. For this reason, the best option was to use 65 °C with 70% of acetone, and the parametric values were 36.2 mg Fx/g AS, and 0.012 min^−1^, respectively. Nevertheless, these conditions are not desirable on an industrial scale because the *r* value was too low and the RMSE-MAE value was 9.43, which was the highest RMSE-MAE value obtained (representing up to 80% of the total value of RMSE-MAE) (Table 2); however, its *R*^2^ value was very good (above 0.991).

Furthermore, high extraction rates indicate a low extractive time (*t*). Equation (2) can be used to calculate *t* at a certain *T* and *S*. When determining the best conditions to extract the maximum amounts of Fx (*Y*_1_), *t* was 61 min at 45 °C, with 70% acetone. Regarding the best conditions to extract the maximum yield (*Y*_2_), *T* = 45 °C, and *t* was ~ 3 min for both *S* = 50% and *S* = 60%. In this case, the *t* for both conditions was very similar; therefore, both examples could be used to obtain desirable conditions. Finally, when analyzing the purity of the extract (*Y*_3_), *t* was 578 min at T = 45 °C and *S* = 70% acetone; however, this value of *t* could be an issue on an industrial level due to the elevated energy costs and slow process associated with it.

Nevertheless, on an industrial scale, industries are usually interested in using extraction processes that require spending the least amount of resources, thus allowing for the best environmental and economic returns. Moreover, this translates to low acetone consumption for minimal production of solvent waste; the use of intermediate temperatures, in which energy consumption is low to moderate; and the shortest extraction times possible. Furthermore, the use of very high temperatures (65 °C) could lead to the evaporation of most of the acetone, as the temperature is set above its boiling point (56 °C), resulting in the reduced effectiveness of the extraction process.

Therefore, industries can adapt conditions to the most desirable parameters in order to obtain the best results with the best economic returns. Depending on time availability, industries can choose conditions in which *t* is slightly higher, as the extracted amount, yield, or purity of Fx remains almost unchanged between different *t*. For example, in the case of *Y*_3_, using *t* = 578 min would obtain the best results, but the energy costs associated with such long extraction times could be very high, which is an environmental and economic disadvantage. With this in mind, the choice of optimal conditions depends on the specific objectives of each industry.

### 3.3. Evaluation of the Antioxidant Response

**Figure 3:**
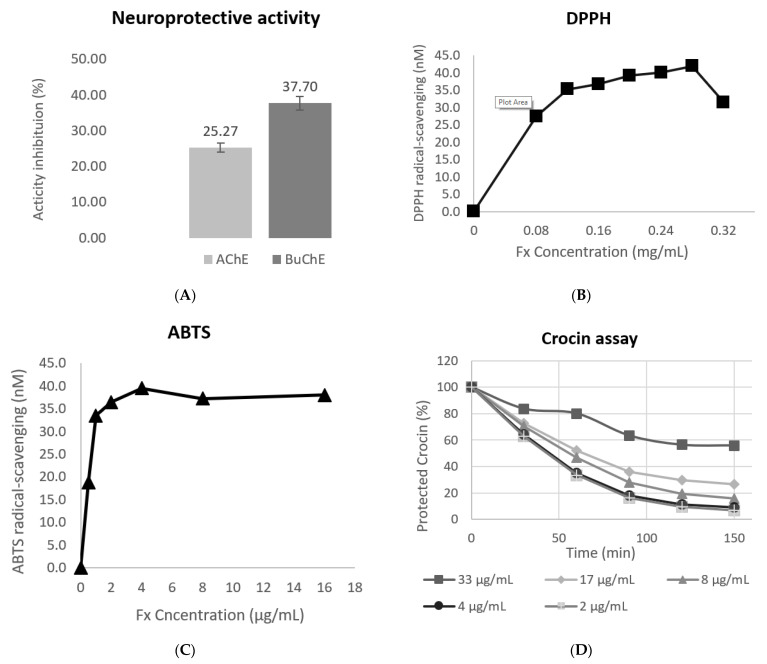
Neuroprotective and antioxidant activity assays. (**A**) Results in % of the inhibition activity of AChE and BuChE using Fx. (**B**) DPPH assay results. (**C**) ABTS assay results. (**D**) Crocin assay results at different times.

The antioxidant activity of the optimal extract was evaluated using three in vitro assays (DPPH and ABTS radical-scavenging activity and inhibition of Crocin bleaching). Several studies have reported on the antioxidant activity of Fx before. The results obtained using DPPH, ABTS, and Crocin (Figure 3) were heterogeneous. In the DPPH assay, the EC50 value obtained was 62.45 µg/mL. Despite being the highest result of the three assays, in previous studies, the result of DPPH was even higher (201 ± 21.4 µg/mL) [23], thus showing worse antioxidant activity than the activity that was observed in the present study. Moreover, in another study, Fx and its derivate, fucoxanthinol, displayed similar results (EC50 164 µM and 153 µM, respectively) to those obtained in this study (95 µM) [22]. Results for the ABTS assay were the best results obtained, with the lowest EC50 being 0.49 µg/mL. Similar studies obtained results up to 85 times higher (33.54 µg/mL) than the results obtained in this study [47]. The Crocin assay results showed the time-dependent antioxidant protection of Fx with an EC50 of 13.47 µg/mL; therefore, the results show that Fx displays antioxidant activity, as was previously reported by other studies [26]. The antioxidant activity of Fx could be attributed to its unique chemical structures that possess an aceryl functional group and an allenic bond [48,49].

### 3.4. Evaluation of the Neuroprotective Activity

Several studies reported the neuroprotective activity of Fx. In this study, two different assays, AChE and BuChE, were used to evaluate the neuroprotective activity of the optimized extract. The studied extract obtained an inhibition activity of 25.27% for the AChE enzyme and 37.70% for the BuChE enzyme (Figure 3). Acetylcholinesterase and butyrylcholinesterase activity are photometric methods wherein the enzyme activities are measured using the rate of production of thiocholine, as both enzymes are hydrolyzed [43].

Alzheimer’s disease is one of the main problems caused by neurodegeneration. It is thought that this disease, and the memory impairments associated with it, may be caused by a cholinergic system defect, which could be counterbalanced with the inhibition of AChE [50]. Similarly, in recent years, butyrylcholinesterase (BuChE) has also been recognized as one of the main agents that could combat neurodegenerative diseases, because the inhibition of this enzyme can increase the levels of acetylcholine in the brain [51]; therefore, inhibition of AChE and BuChE would result in boosting the cholinergic system, which prompts us to consider that the inhibition of these two enzymes could have a positive effect in combating Alzheimer’s disease and other neurodegenerative illnesses; thus, the extract that was used could have a neuroprotective effect.

These results are supported by the literature, with regard to the in vivo studies that were performed. Zhang et al., 2017, obtained positive results when studying the neuroprotective proprieties of Fx in models of traumatic brain injury (TBI) and the role of the nuclear factor erythroid 2-related factor 2 (Nrf2)-antioxidant-response element (ARE) and Nrf2-autophagy pathways in putative neuroprotection. The motor performance of Fx-treated mice was significantly better than the performance of the vehicle-treated mice after one day [24].

Additionally, other authors have tested the administration of Fx to determine if it could attenuate cerebral ischemic/reperfusion (I/R) injury, and they tried to elucidate its possible mechanisms using an in vivo middle cerebral artery occlusion (MCAO) model and an in vitro oxygen–glucose deprivation and reoxygenation (OGD/R) model. Their findings suggested that Fx could be exploited as a therapeutic target for protecting neurons [52].

Furthermore, oxidative stress also plays an important role in the acceleration of neurodegenerative diseases; therefore, the use of compounds such as Fx, which have antioxidant properties, similarly to the previously mentioned compounds, will also provide a synergic effect, and improve the neuroprotection provided. In vitro studies also demonstrated that Fx increased neuron survival and reduced the level of reactive oxygen species (ROS) [24].

### 3.5. Evaluation of the Antimicrobial Activity

The antimicrobial assay shows that *Bacillus cereus, Staphylococcus aureus*, and *Pseudomonas aeruginosa* presented inhibitions in terms of their growth, as a result of Fx. *B. cereus* and *S. aureus* were the bacterial strains which presented higher growth inhibition, at 8.99 ± 0.22 and 10.40 ± 1.29 mm, respectively. *P. aeruginosa* presented an inhibitory halo of 5.44 ± 0.35 mm, and the rest of the species (*E. coli, S. epidermis*, *S. enteridis*) did not present inhibitory activity as a result of Fx.

In previous studies, the three bacteria strains also showed growth inhibition, as a result of Fx, with comparable results [53,54]. Nevertheless, the concentration of Fx used in previous studies was lower than the one used in this study (previous studies had a concentration of 1 mg/mL and 0.1 mg/mL, vs. this study, which had a concentration of 20 mg/mL). Nonetheless, the extraction methods used were different, which influences the composition of the final tested extract, and this could affect the antimicrobial activity of Fx. Moreover, in these studies, *E. coli, S. epidermidis*, and other species of the genus *Salmonella*, presented an inhibition halo during the disk diffusion test [54]; however, the result obtained still shows that Fx has the capacity to inhibit the growth of both gram-positive and gram-negative bacteria.

### 3.6. Validation of the Biological Properties through In Silico Studies

#### 3.6.1. Molecular Docking

To analyze the results of the different bioactivities exhibited by Fx, five proteins related to antibacterial and neuroprotective activity were chosen to try and find the interactions that produce these bioactivities. Proteins KS, GY, and DR are related to different microbial activities that make them target antimicrobial agents. KS is implicated in the synthesis of Type II fatty acids. Fatty acids are critical for bacteria; therefore, inhibition of KS, which is directly implicated in fatty acid elongation, could imply that it impedes bacterial cell development [55,56]. GY belongs to the Type II subdivision of topoisomerases that play a crucial role in maintaining DNA conformation during DNA replication. This important role makes them a target for several antimicrobials [57,58]. DR is a well-known target of antibiotics as a result of its effects during the synthesis of purines, thymidylate, and some amino acids [59].

On the other hand, AChE and BuChE have a close relationship with neurodegenerative diseases, specifically with Alzheimer’s disease. AChE inhibitors are usually used in the dementia phase; donepezil, rivastigmine, galantamine, and the glutamate antagonist, memantine, are the most used inhibitors. These drugs improve the synaptic levels of acetylcholinesterase and increase the cholinergic function in the brain [60,61,62]. Accordingly, a new inhibitor of AChE could be a potential drug to fight against Alzheimer´s disease. BuChE levels, in advanced stages of Alzheimer´s disease, show increased values of up to 120% compared with their normal values [63]. This increase in BuChE levels could be related to a possible function of this protein in the progression of Alzheimer´s disease, and that is the reason why BuChE is becoming a new target for neuroprotective drugs [64].

For the validation of the molecular docking methodology, the RMSD was calculated between the coordinates of the ligands that were complexed with the selected proteins, and the coordinates that had the best affinity after molecular docking. The RMSD between these two molecules was calculated in PYMOL with the command “align “ligand before docking*, ligand after docking*, cycles = 0, transform = 0”. The results showed that all of the simulated positions had an RMSD between 0.079 and 0.152 Å, compared with the experimental positions of the complexed ligands; this fact makes the in silico study more robust.

**Figure 4:**
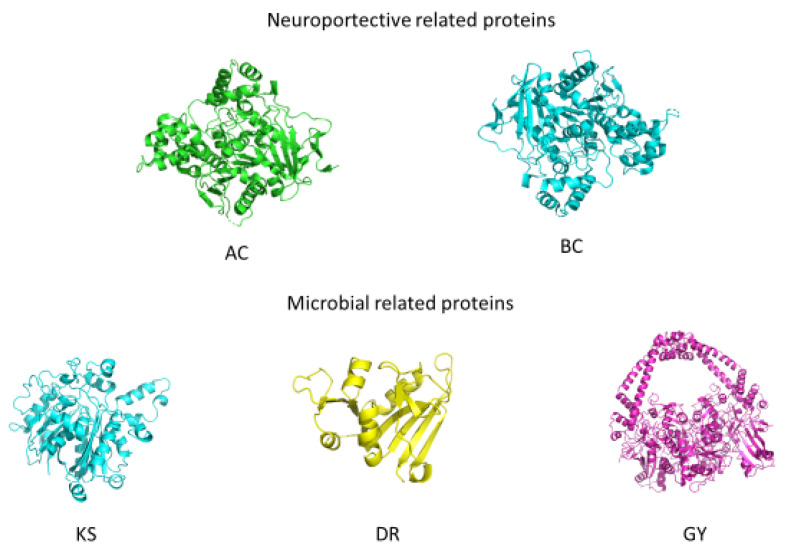
Three-dimensional structures of the docked proteins.

The results obtained in the experimental assays promoted the performance of molecular docking, with molecules related to antimicrobial and neuroprotective activities, which produced significant results. The structures of the proteins selected for molecular docking can be seen in Figure 4.

Table 4 shows the principal characteristics of the proteins and the results of the Ramachandran plot, which showed that all the proteins have more than 88% of amino acids in their most favorable regions, thus indicating a good protein structure [65]. The RMSD obtained between the simulated donepezil coordination and the download coordination was 0.238 Å.

The results obtained after docking has occurred between Fx and the proteins under study are shown in Table 5.

**Figure 5:**
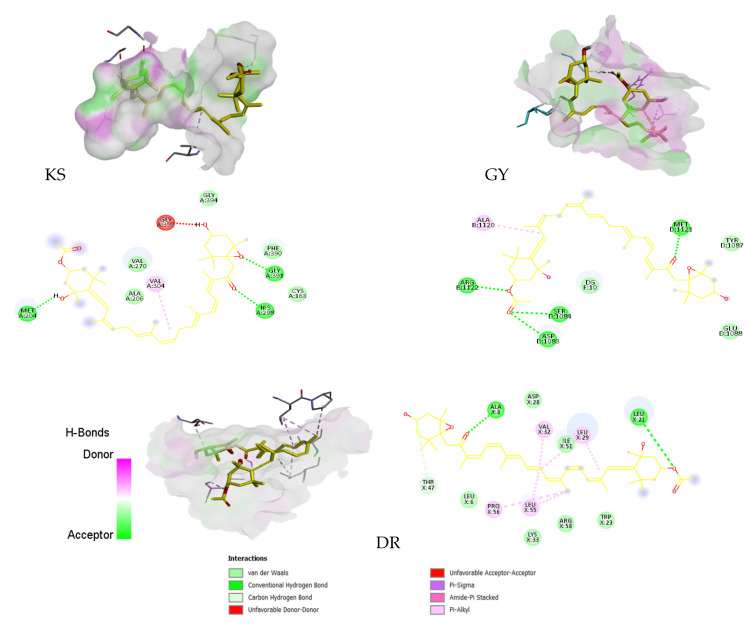

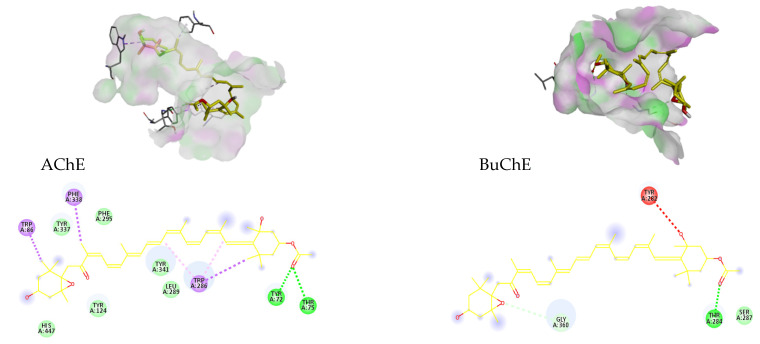
Representation of the molecular interactions between Fx and beta-ketoacyl-ACP I synthase, dihydrofolate reductase, gyrase acetylcholinesterase, and butyrylcholinesterase after docking. In yellow, the integration of Fx with the protein’s active center can be seen. In violet, the donor regions of hydrogen bonds can be seen, and in green, the accepters can be seen. We can also observe the distinct types of interactions between the residues involved in the molecular junction (represented by dashed lines in different colors according to each type).

The results obtained show that Fx has a high affinity for AChE and DR. In the case of proteins that are related to antibacterial activity, Fx had a high affinity for DR. After docking occurred between Fx and DR, the combination of the two hydrogen bonds (ALA-8 and LEU-21), and the bonding forces produced by alkane interactions, resulted in a binding affinity of 8.1 kcal/mol that represents a *k_i_* below 0.1 M, which denotes a high affinity for the active center of DR (Figure 5). In previous studies, it was found that both ALA-8 and LEU-21 are related to the interactions between different DR inhibitors [66,67]; therefore, Fx could be a potential DR inhibitor. Concerning these results, the antibacterial activity of Fx has recently been evaluated, and positive results were obtained in Gram-positive bacteria, and a lower median was found in Gram-negative bacteria [54]. Given that the protein that has a higher affinity with Fx (DR) is from *Staphylococcus aureus*, these results corroborate the results obtained in the disk diffusion test; therefore these results suggest that Fx has potential antibacterial activity [54,68].

Results obtained in relation to the proteins used as a target for the inhibition of neurodegenerative disease development, show that Fx has a high affinity for AChE (Table 5). Fx formed two H-B with the aa TYR72 and THR 75. These two residues are involved in the formation of H-B with different protein inhibitors [69,70] In previous studies, Fx has been linked to numerous neuroprotective processes in different neurodegenerative pathologies [24,71,72]. In addition, Fx directly inhibited AChE through a non-competitive mechanism in vitro. This inhibition may have been caused by the actions of Fx at the peripheral anionic site of acetylcholinesterase [73]. The results obtained corroborate the results of previous studies, and reaffirm Fx as a potential alternative for treating neurodegenerative diseases such as Alzheimer’s. On the other hand, Fx did not present a high affinity for BuChE. Nevertheless, the results obtained in the docking do not concur with the results obtained in the neuroprotective activity assay, where FX inhibits the BuChE activity more efficiently than the AChE activity.

Given the high affinity obtained between Fx and the active center of acetylcholinesterase, this pigment could be used as a molecular alternative for the acetylcholinesterase inhibitor drugs that are currently being used (Table 5). The results show that the compound with the best affinity for the active center of acetylcholinesterase is donepezil, with a *k_i_* of 2.2 nM. Moreover, Fx was the compound with the second-best affinity, with a *k_i_* of 3.1 nM. Comparing Fx with the rest of the compounds gives a result that is very close to that obtained by the donepezil, and it is superior to the other three inhibitors tested; therefore, and as stated above, the results reaffirm the neuroprotective potential of Fx in the treatment of different neurodegenerative pathologies such as Alzheimer’s.

#### 3.6.2. Pharmacokinetic Study

Among other parameters and predictions, SwissADME predicts five key pharmacokinetic behaviors when designing a drug. The parameters predicted by SwissADME are: gastrointestinal absorption (GI), blood–brain barrier permeability (BBB), glycoprotein-P interactions (Pgp), cytochrome P interactions (CYP), and permeability coefficient (*k*_p_) [46,74].

SwissADME results show that Fx can be absorbed by intestinal epithelial cells, but to a small extent. As for the penetration of the blood–brain barrier, Fx does not display any attributes that indicate it is able to pass over without being modified or coupled to other molecules. Nevertheless, another ADME in silico study found high BBB penetration [75]. Given their negative CYP result, it is not toxic to these proteins. On the other hand, it tested positive in Pgp proteins; therefore, they could potentially form a substrate which is able to cause problems when the compound tries to penetrate the intracellular matrix. The high value of *k_p_* that was obtained, indicates poor permeability on the part of Fx. These results are all shown in Table 6.

Despite these results, some studies found that Fx has favorable drug-like properties [75]; however, other studies found that Fx is mainly accumulated, similarly to astaxanthin, in the liver, heart, and adipose tissues [76].

## 4. Conclusions

Fx is considered a valuable molecule due to its wide range of beneficial properties, such as its antioxidant, anti-inflammatory, and antimicrobial activities, as well as its neuroprotective effects. Thus, Fx is a carotenoid of interest for the industry, and the kinetic behavior of Fx extraction has become more significant in the last decade. In this study, Fx was obtained from *U. pinnatifida* with acetone, using a CHE. A first-order kinetic equation, structured in two parameters, was used to fit the experimental data. The kinetic parameters were *r* (min^−1^), which is related to the extraction rate, and *k* (*µg/g*), which represents the maximum amounts of Fx that can be extracted (*Y*_1_). Moreover, *k*_2_ (*mg/g*) is related to the yield (*Y*_2_), and *k*_3_ (*mg/g*) is related to the purity of the extract (*Y*_3_). The highest possible values of the parameters *k* and *r* were the main standards used to find the optimal conditions for extraction; in other words, the maximum amounts of Fx that can be obtained at the highest possible extractive rates. Based on the best conditions for *T* and *S*, the optimal extraction time (*t*) was calculated from *r*. The optimal conditions for *Y*_1_ were *T* = 45 °C, *S* = 70%, and *t* = 61 min, and the response was ~ 5024 µg Fx/g AS dw; for *Y*_2_ the optimal conditions were *T* = 45 °C, *S* = 50% and 60%, and *t* = ~3 min, and its response was ~ 465 mg E/g AS dw; and for *Y*_3_ the optimal conditions were *T* = 45 °C, *S* = 70%, and *t* = 578 min and its response was 11.2 mg Fx/g E dw. Additionally, the fitting errors of the kinetic equation with the experimental data were relatively small (low values of RMSE-MAE) for all *Y*; therefore, it can be stated that the model fits the experimental data with good reliability. Moreover, these results show that the Fx molecule presents a more robust stability than what is described in the literature, as it resists quite high temperatures, such as 65 °C, for several hours, without suffering degradation.

Furthermore, this work also confirms the findings in the literature as the *EC*_50_ values for the DPPH assay were 62.45 µg/mL, 0.49 µg/mL for the ABTS assay, and the crocin discoloration was determined at an *EC*_50_ of 13.47 µg/mL. These results show an antioxidant activity of Fx that is higher than has been previously reported by other studies. Furthermore, the Fx extract showed an inhibition activity of 25.27% for the AChE enzyme and 37.70% for the BuChE enzyme, which prompts us to consider that Fx could have a positive effect in combating neurodegenerative illnesses, and that the extract that was used has a neuroprotective effect. Additionally, the extract showed antimicrobial activity against three of the bacterial species evaluated, namely, the gram-negative bacteria *Pseudomonas aeruginosa*, and the gram-positive bacteria *Bacillus cereus* and *Staphylococcus aureus*.

The positive results for the neuroprotective and antimicrobial activities of Fx prompted an in silico study of molecular docking between proteins that are related to both activities, as well as a study on the pharmacokinetic characteristics of Fx.

These bioactivities turn Fx into an interesting pigment with promising industrial applications in the food, cosmetic, and pharmaceutical sectors. Nevertheless, the commercialization of Fx is scarce, which limits its further use. Fx may be chemically synthesized, but this process is inefficient and complex, and the extraction method from marine organisms has not been standardized; therefore, it was necessary to design a practical way to profit from its properties. The application of methods such as these, is likely to optimize the extraction conditions for Fx. Thus, future, innovative studies regarding efficient, quick, eco-friendly, and safe extraction methods can speed up the progress towards the commercialization and incorporation of Fx in the global market.

## Figures and Tables

**Table 1:** The parametric values, *k* (µg/g) and *r* (min^−1^), at different temperatures (*T*) and acetone concentrations (*S*). In addition, *R*^2^ values for different temperatures and acetone proportions are shown.

Variables		*Y*_1_ (µg Fx/g AS)					*Y*_2_ (mg E/g AS)					*Y*_3_ (mg Fx/g E)				
*T*	*S*	*k*		*r*		*R* ^2^	*K*		*r*		*R* ^2^	*k*		*r*		*R* ^2^
(°C)	(%)	(µg Fx/g AS)		(min^−1^)			(mg E/g AS)		(min^−1^)			(mg Fx/g E)		(min^−1^)		
**5**	**50**	191	ns	0.295	ns	0.6120	444	±295.0	0.212	ns	0.9765	0.6	ns	0.261	ns	0.5024
	**60**	2708	±297.6	0.048	±0.036	0.9825	407	±297.6	0.080	±0.036	0.9825	6.4	±6.4	0.073	ns	0.9825
	**70**	4618	±274.5	0.037	±0.015	0.9731	487	±274.5	0.043	±0.015	0.9731	11.2	±5.8	0.050	ns	0.9731
	**80**	4227	±282.3	0.025	±0.011	0.9657	462	±282.3	0.041	±0.011	0.9657	10.8	±5.9	0.038	ns	0.9657
	**90**	2407	±343.6	0.008	±0.006	0.9441	172	Ns	0.070	±0.006	0.9441	12.7	±6.9	0.020	ns	0.9441
	**100**	2887	±504.0	0.001	±0.001	0.8202	38	Ns	0.005	±0.001	0.8202	56.0	±6.4	0.090	ns	0.8202
**25**	**50**	415	±330.6	0.065	ns	0.8708	407	±330.6	0.332	ns	0.9766	1.1	ns	0.463	ns	0.9595
	**60**	3956	±381.7	0.051	±0.020	0.9912	403	±381.7	0.319	±0.020	0.9912	9.8	±8.2	0.055	ns	0.9912
	**70**	4240	±292.6	0.065	±0.024	0.9978	413	±292.6	0.246	±0.024	0.9978	10.2	±6.3	0.067	ns	0.9978
	**80**	4206	±296.5	0.047	±0.016	0.9948	391	±296.5	0.402	±0.016	0.9948	10.7	±6.4	0.050	ns	0.9948
	**90**	1809	±331.5	0.048	±0.039	0.9341	154	Ns	0.309	±0.039	0.9341	11.7	±7.1	0.049	ns	0.9341
	**100**	2751	±393.5	0.009	±0.005	0.7484	36	Ns	0.033	±0.005	0.7484	70.1	±7.1	0.049	±0.023	0.7484
**45**	**50**	189	ns	0.260	ns	0.9120	468	±401.0	2.486	±2.107	0.9959	0.3	ns	1.915	ns	0.8117
	**60**	4674	±428.6	0.176	±0.057	0.9787	463	±428.6	2.129	±0.057	0.9787	7.0	ns	1.069	ns	0.9787
	**70**	5029	±326.5	0.113	±0.032	0.9594	463	±326.5	0.460	±0.032	0.9594	10.5	±6.9	0.156	ns	0.9594
	**80**	3667	±330.7	0.093	±0.037	0.9862	444	±330.7	0.152	±0.037	0.9862	8.1	±6.4	0.368	ns	0.9862
	**90**	1968	±337.2	0.304	±0.233	0.8638	124	Ns	0.476	±0.233	0.8638	14.6	±6.4	1.543	ns	0.8638
	**100**	4063	±386.8	0.024	±0.010	0.7838	34	Ns	0.036	±0.010	0.7838	109.2	±5.8	4.979	ns	0.7838
**65**	**50**	347	ns	0.872	ns	0.9584	428	±419.4	3.008	ns	0.9985	0.8	ns	2.673	ns	0.9482
	**60**	3274	±338.5	0.297	±0.173	0.9909	437	±338.5	1.990	±0.173	0.9909	6.3	ns	3.434	ns	0.9909
	**70**	3914	±290.9	0.162	±0.058	0.9906	380	±290.9	2.373	±0.058	0.9906	36.2	±12.8	0.012	±0.011	0.9906
	**80**	2381	±274.3	0.289	±0.174	0.9708	406	±274.3	0.807	±0.174	0.9708	5.7	ns	0.446	ns	0.9708
	**90**	2597	±405.1	0.249	±0.147	0.8919	141	Ns	0.300	±0.147	0.8919	17.5	±7.5	1.005	ns	0.8919
	**100**	3174	±363.6	0.045	±0.020	0.7444	31	Ns	0.234	±0.020	0.7444	105.4	±7.3	0.065	±0.019	0.7444

Abbreviations: ns: not statistical significant; *R*^2^: coefficient of determination; Fx: Fucoxanthin; AS: Alga Sample; E: Extract.

**Table 2:** MAE, RMSE, and RMSE-MAE values for each response (*Y*) at different *T* and *S*.

Variables		*Y*_1_ (µg Fx/g AS)			*Y*_2_ (mg E/g AS)			*Y*_3_ (mg Fx/g E)		
*S*	*T*	MAE	RMSE	RMSE-MAE	MAE	RMSE	RMSE-MAE	MAE	RMSE	RMSE-MAE
(%)	(°C)									
**50**	**5**	43.95	53.29	9.34	17.86	24.10	6.25	0.20	0.23	0.03
	**25**	44.55	58.49	13.94	18.07	23.48	5.42	0.06	0.08	0.02
	**45**	16.16	20.74	4.58	9.13	11.20	2.07	0.05	0.06	0.01
	**65**	22.15	28.43	6.28	4.81	6.61	1.80	0.07	0.07	0.01
**60**	**5**	189.55	265.20	75.65	10.97	15.88	4.91	0.74	0.97	0.23
	**25**	371.33	444.06	72.73	10.02	11.63	1.62	0.97	1.24	0.27
	**45**	264.95	343.90	78.95	5.17	6.32	1.14	1.40	1.80	0.40
	**65**	319.24	440.00	120.76	7.46	8.74	1.28	1.21	1.37	0.16
**70**	**5**	263.78	388.14	124.36	48.32	146.04	97.73	0.66	0.94	0.28
	**25**	256.06	354.93	98.87	4.90	7.04	2.13	0.68	0.93	0.25
	**45**	364.91	483.49	118.58	16.31	23.13	6.82	0.79	1.24	0.45
	**65**	164.07	363.35	199.29	32.29	118.69	86.40	4.17	19.21	15.04
**80**	**5**	244.76	356.14	111.38	41.53	123.47	81.94	0.53	1.12	0.59
	**25**	324.49	440.27	115.77	6.99	10.52	3.53	0.74	1.03	0.30
	**45**	460.77	676.96	216.19	32.58	45.80	13.22	0.84	1.32	0.49
	**65**	198.90	484.48	285.58	4.84	13.54	8.70	0.43	0.75	0.32
**90**	**5**	415.83	499.51	83.68	11.53	15.45	3.92	1.87	2.26	0.39
	**25**	189.80	223.55	33.75	4.93	6.94	2.01	0.90	1.18	0.28
	**45**	206.90	239.23	32.32	6.73	9.32	2.59	1.01	1.57	0.56
	**65**	119.74	154.98	35.24	2.71	3.27	0.56	0.71	0.86	0.15
**100**	**5**	464.49	546.32	81.83	4.50	5.65	1.15	10.37	14.89	4.51
	**25**	353.15	403.64	50.49	4.57	5.76	1.18	10.78	13.25	2.47
	**45**	628.71	825.88	197.17	4.71	6.46	1.75	10.56	14.92	4.36
	**65**	274.31	429.70	155.39	2.79	6.19	3.40	17.38	25.67	8.30

**Table 3:** Trend and dependence of the kinetic parameters (*k* and *r*) on *T* and *S*.

***Y*_1_ (µg Fx/g AS)**			
Same solvent	*k*	constant	does not depend on T
	*r*	increases with T	depends on T
Same temperature	*k*	increases and decreases with S (curve)	depends on S
	*r*	decreases with S	depends on S
***Y*_2_ (mg E/g AS)**			
Same solvent	*k*	constant	does not depend on T
	*r*	increases with T	depends on T
Same temperature	*k*	decreases with S	depends on S
	*r*	decreases with S	depends on S
***Y*_3_ (mg Fx/g E)**			
Same solvent	k	constant	does not depend on T
	r	not statistically significant *	-
Same temperature	*k*	increases with S	depends on S
	*r*	not statistically significant *	-

Note: * due to a considerable lack of statistically significant data, the trend and dependence of the kinetic parameters were not determined.

**Table 4:** Characteristics of the proteins selected for molecular docking.

Protein	PDB ID	Organism	R	Rp	Ligand Complex
AChE	4EY7	*Homo sapiens*	2.35 Å	90.3%	Donepezil
BuChE	1P0P	*Homo sapiens*	2.30 Å	88.2%	N-acetylglucosamine
KS	1FJ4	*Escherichia coli*	2.35 Å	88.4%	Thiolactomycin
GY	2XCS	*Staphylococcus aureus*	2.10 Å	92.2%	GSK-299423
DR	3SRW	*Staphylococcus aureus*	1.70 Å	89.7%	Q27

R; resolution of the protein structure, Rp; Ramachandran plot.

**Table 5:** Results of Fx docking with proteins that are related to antimicrobial and neuroprotective properties and AChE inhibitors.

Protein/Inhibitor	H-B	Be (kcal/mol)	k_i_ (μM)	AA with H-B Interactions
**Antimicrobial**				
**KS**	3	−8.1	1.155	MET 204, HIS 298, GLY 391
**GY**	4	−7.5	3.180	GLN 91, SER 128, ASP 81
**DR**	2	−9.7	0.078	ALA 8, LEU 21
**Neuroprotective**				
**AChE**	2	−11.6	0.003	TYR 72, THR 75
**BuChE**	1	−6	39.991	THR 284
**AChE Inhibitors**				
**Donepezil**	1	−11.8	0.0022	SER125
**Fx**	2	−11.6	0.0031	TYR 72, THR 75
**Galanyamine**	0	−8.7	0.4196	-
**Memantine**	0	−8.1	1.1552	-
**Rivastigmine**	1	−8	1.3676	PHE 295

H-B; hydrogen bond, *k_i_*; inhibition constant, Be; binding energy.

**Table 6:** Results of the pharmacokinetic study and certain molecular characteristics of Fx.

Formula	C42H58O6	Pgp substrate	Yes
Molecular weight	658.91 g/mol	CYP1A2 inhibitor	No
Heavy atoms	48	CYP2C19 inhibitor	No
H-B acceptors	6	CYP2C9 inhibitor	No
H-B donors	2	CYP2D6 inhibitor	No
GI	Low	CYP3A4 inhibitor	No
BBB	No	log *Kp* (cm/s)	−460

GI; gastrointestinal absorption, P-H; hydrogen bonds, BBB; blood–brain barrier permeability, Pgp; interactions with glycoprotein-P, CYP; interaction with cytochrome P, *K_p_*; permeability coefficient.

## Data Availability

Data is contained within the article.

## Abbreviations

***Generic***	
ARE	Antioxidant-response element
E	Extract
*EC*_50_	Half maximal effective concentration
Fx	Fucoxanthin
CHE	Convectional heat extraction
HPLC-DAD	High Performance Liquid Chromatography - Photodiode array detector
AS dw	Algae sample dry weight
MAE	Mean absolute error
PDB	Protein Data Bank
RMSE	Root mean squared error
ROS	Reactive oxygen species
SMILES	Simplified Molecular Input Line Entry
*S*	Solvent concentration
*t*	Time
*T*	Temperature
UV	Ultraviolet
***Compounds***	
AAPH	2,2′-azobis-2-amidinopropane: RN = NR
ABTS	2,2′-azino-bis(3-ethylbenzothiazoline-6-sulfonic acid
ALA	Alanine
DMSO	Dimethyl sulfoxide
DNA	Deoxyribonucleic acid
DPPH	2,2-diphenyl-1-picrylhydrazyl
HCl	Hydrochloric acid
LEU	Leucine
K_2_S_2_O_8_	Potassium persulfate
THR	Threonine
TYR	Tyrosine
***Neuroprotective activity***	
I/R	Ischemic/reperfusion
MCAO	Middle cerebral artery occlusion
MHB	Mueller Hinton Broth
Nrf2	Nuclear factor erythroid 2-related factor 2
Nrf2-ARE	Nuclear factor erythroid 2-related factor 2 antioxidant-response element
OGD/R	Oxygen-glucose deprivation and reoxygenation
TBI	Traumatic brain injury
***Proteins***	
AChE	Acetylcholinesterase
BuChE	Butyrylcholinesterase
DR	Dihydrofolate reductase
GY	Gyrase
KS	Beta-ketoacyl-(acyl carrier protein) synthase I
***In silico studies***	
ADME	Absorption, distribution, metabolism and excretion
BBB	Blood–brain barrier permeability
CYP	Cytochrome P interaction
GI	Gastrointestinal absorption
*K_p_*	Permeability coefficient
Pgp	Glycoprotein-P interaction
RMSD	Root-mean-square deviation

## Appendix A

**Table A1:** Kinetic results of Fx extraction as a function of solvent concentration (S, %), time (*t*, min) and extraction temperature (*T*, °C). Responses are expressed as: *Y*_1_, fucoxanthin per gram of alga sample (mg Fx/g AS dw); *Y*_2_, extract yield, or mg extract per gram of alga sample (mg E/g AS dw).

Extraction Variables		Extraction Responses for Acetone Solvent in Different Concentrations											
		*Y*_1_						*Y*_2_					
*T* (°C)	*t* (min)	50%	60%	70%	80%	90%	100%	50%	60%	70%	80%	90%	100%
5	30	239.2	2057.4	3193.6	2533.7	1016.0	668.5	433.7	369.7	363.3	335.3	143.9	12.0
	120	297.2	2840.7	3790.7	3322.0	1405.9	1034.7	390.5	379.7	382.6	369.9	143.9	24.4
	480	137.8	3228.9	4298.1	3844.8	1530.0	1190.8	470.3	415.0	439.7	397.9	173.3	35.3
	1200	157.0	2637.1	5165.2	4196.8	2016.7	1597.6	452.4	400.4	433.7	397.2	167.3	28.6
	2640	167.0	2524.3	4487.8	4464.3	2863.3	2195.7	461.0	437.0	413.8	425.2	189.3	36.6
	4200	149.2	1871.2	4846.9	4546.0	3075.2	3679.3	456.4	402.4	383.3	394.6	194.6	43.3
	5700	114.5	2043.8	4975.1	4550.9	3890.6	3673.2	431.1	425.7	447.0	348.7	201.2	48.6
	9680	79.8	2305.4	4957.5	4594.8	4418.8	5318.1	471.7	424.3	412.5	429.2	203.9	51.3
25	15	462.1	2511.4	2938.1	2500.1	1114.6	1004.1	427.5	393.4	398.0	392.6	154.4	23.9
	30	355.6	2692.6	3244.5	2830.2	1204.1	1148.4	358.9	388.0	414.7	368.6	151.1	23.2
	120	507.7	3284.8	3881.2	3394.3	1415.6	1396.8	423.5	421.3	419.3	389.9	146.4	26.6
	480	455.7	4081.2	4482.4	4495.1	1730.4	2306.9	421.5	414.0	425.9	406.6	148.4	41.8
	1200	375.5	4593.3	4773.3	4925.0	2095.6	2995.2	399.6	393.4	402.0	396.6	155.1	31.9
	1680	645.5	3915.4	4466.6	4275.9	2022.4	3055.0	423.5	415.3	416.0	382.6	169.1	38.5
	2640	321.8	3415.6	3746.5	3981.9	1735.8	3625.9	432.9	398.0	406.7	397.3	177.1	41.2
45	3	118.6	2118.9	1693.3	1238.7	1329.4	1231.9	480.8	465.2	318.4	160.3	98.9	11.3
	5	127.0	2932.8	2812.3	1881.1	1418.4	1408.4	458.9	459.2	460.7	272.8	112.9	13.3
	15	153.4	3620.0	3266.8	2190.6	1613.6	1706.3	466.2	469.2	443.4	345.9	110.9	20.0
	60	194.1	4881.1	4800.9	2431.3	1712.9	2375.1	481.4	469.2	468.0	363.2	116.9	18.6
	210	185.0	2456.4	4218.6	3383.3	2179.1	2725.0	449.6	451.2	454.7	458.4	120.8	30.6
	1200	161.2	4761.4	5565.2	4418.8	2018.8	4675.5	452.2	452.6	487.9	517.6	142.8	36.6
	1680	223.3	4934.7	5465.5	4571.4	1998.9	4960.0	417.7	436.6	443.4	465.0	138.1	41.3
	2640	208.2	3689.9	4621.5	4616.4	2345.1	4739.4	444.2	442.6	341.7	466.4	151.4	51.3
65	3	315.6	2062.8	1803.4	1559.7	1507.3	1038.4	432.9	433.4	402.9	369.0	86.5	17.3
	5	364.7	2325.0	2219.5	1718.7	1771.6	1180.1	415.5	448.1	438.1	398.9	107.1	23.2
	15	297.4	2300.0	3095.0	1801.6	2286.3	1647.0	427.5	428.7	440.8	400.2	141.0	29.2
	30	381.1	3669.6	3753.6	2223.0	2580.7	2050.3	435.1	448.3	457.6	398.2	146.3	33.2
	60	178.7	3434.1	3417.4	1688.0	2548.4	2479.6	430.6	425.0	390.0	378.3	136.3	25.4
	120	111.1	1673.1	3882.6	2267.8	2497.9	2740.9	450.6	441.6	437.6	407.1	154.1	17.7
	210	202.2	3566.7	4101.7	2830.6	2834.1	3390.1	405.1	410.6	51.0	423.7	154.1	37.6
	480	298.5	3221.8	4671.2	3405.5	1581.2	3815.0	420.9	441.4	416.8	426.8	153.0	40.5
	1200	43.2	1995.0	808.9	4379.2	1814.0	4336.8	430.2	388.1	419.5	440.3	174.0	43.1

## References

[1] Lourenço-Lopes C., Garcia-Oliveira P., Carpena M., Fraga-Corral M., Jimenez-Lopez C., Pereira A.G., Prieto M.A., Simal-Gandara J. (2020). Scientific approaches on extraction, purification and stability for the commercialization of fucoxanthin recovered from brown algae. Foods.

[2] Saet B.L., Joo Y.L., Song D.G., Pan C.H., Chu W.N., Min C.K., Eun H.L., Sang H.J., Kim H.S., Yeong S.K. (2008). Cancer chemopreventive effects of Korean seaweed extracts. Food Sci. Biotechnol..

[3] Lourenço-Lopes C., Fraga-Corral M., Jimenez-Lopez C., Pereira A.G., Garcia-Oliveira P., Carpena M., Prieto M.A., Simal-Gandara J. (2020). Metabolites from macroalgae and its applications in the cosmetic industry: A circular economy approach. Resources.

[4] Willstatter R., Page H. (1914). The pigments of the brown algae. Justus Liebigs Ann. Chem..

[5] Billakanti J.M., Catchpole O.J., Fenton T.A., Mitchell K.A., Mackenzie A.D. (2013). Enzyme-assisted extraction of fucoxanthin and lipids containing polyunsaturated fatty acids from *Undaria pinnatifida* using dimethyl ether and ethanol. Process Biochem..

[6] Kajikawa T., Okumura S., Iwashita T., Kosumi D., Hashimoto H., Katsumura S. (2012). Stereocontrolled total synthesis of fucoxanthin and its polyene chain-modified derivative. Org. Lett..

[7] Asai A., Sugawara T., Ono H., Nagao A. (2004). Biotransformation of fucoxanthinol into amarouciaxanthin a in mice and HepG2 cells: Formation and cytotoxicity of fucoxanthin metabolites. Drug Metab. Dispos..

[8] Sangeetha R.K., Bhaskar N., Divakar S., Baskaran V. (2010). Bioavailability and metabolism of fucoxanthin in rats: Structural characterization of metabolites by LC-MS (APCI). Mol. Cell. Biochem..

[9] Yamamoto K., Ishikawa C., Katano H., Yasumoto T., Mori N. (2011). Fucoxanthin and its deacetylated product, fucoxanthinol, induce apoptosis of primary effusion lymphomas. Cancer Lett..

[10] Sun P., Wong C.C., Li Y., He Y., Mao X., Wu T., Ren Y., Chen F. (2019). A novel strategy for isolation and purification of fucoxanthinol and fucoxanthin from the diatom *Nitzschia laevis*. Food Chem..

[11] Heo S.J., Ko S.C., Kang S.M., Kang H.S., Kim J.P., Kim S.H., Lee K.W., Cho M.G., Jeon Y.J. (2008). Cytoprotective effect of fucoxanthin isolated from brown algae *Sargassum siliquastrum* against H2O2-induced cell damage. Eur. Food Res. Technol..

[12] D’Orazio N., Gemello E., Gammone M.A., De Girolamo M., Ficoneri C., Riccioni G. (2012). Fucoxantin: A treasure from the sea. Mar. Drugs.

[13] Soo-Jin You-Jin H., Seok-Chun K., Sung-Myung K., Hahk-Soo K., Jong-Pyung K., Soo-Hyun K., Ki-Wan L., Man-Gi C. (2008). Jeon Cytoprotective effect of fucoxanthin isolated from brown algae *Sargassum siliquastrum* against H_2_O_2_-induced cell damage. Eur. Food Res. Technol..

[14] Kumar S.R., Hosokawa M., Miyashita K. (2013). Fucoxanthin: A marine carotenoid exerting anti-cancer effects by affecting multiple mechanisms. Mar. Drugs.

[15] Zhang H., Tang Y., Zhang Y., Zhang S., Qu J., Wang X., Kong R., Han C., Liu Z. (2015). Fucoxanthin: A Promising Medicinal and Nutritional Ingredient. Evidence-Based Complement. Altern. Med..

[16] Van Chuyen H., Eun J.B. (2017). Marine carotenoids: Bioactivities and potential benefits to human health. Crit. Rev. Food Sci. Nutr..

[17] Raguraman V., Abraham S., MubarakAli D., Narendrakumar G., Thirugnanasambandam R., Kirubagaran R., Thajuddin N. (2018). Unraveling rapid extraction of fucoxanthin from *Padina tetrastromatica*: Purification, characterization and biomedical application. Process Biochem..

[18] Wang L., Park Y.J., Jeon Y.J., Ryu B.M. (2018). Bioactivities of the edible brown seaweed, *Undaria pinnatifida*: A review. Aquaculture.

[19] Fung A., Hamid N., Lu J. (2013). Fucoxanthin content and antioxidant properties of *Undaria pinnatifida*. Food Chem..

[20] Liu C.L., Liang A.L., Hu M.L. (2011). Protective effects of fucoxanthin against ferric nitrilotriacetate-induced oxidative stress in murine hepatic BNL CL.2 cells. Toxicol. Vitr..

[21] Wang X., Cui Y.J., Qi J., Zhu M.M., Zhang T.L., Cheng M., Liu S.M., Wang G.C. (2018). Fucoxanthin Exerts Cytoprotective Effects against Hydrogen Peroxide-induced Oxidative Damage in L02 Cells. Biomed Res. Int..

[22] Sachindra N.M., Sato E., Maeda H., Hosokawa M., Niwano Y., Kohno M., Miyashita K. (2007). Radical scavenging and singlet oxygen quenching activity of marine carotenoid fucoxanthin and its metabolites. J. Agric. Food Chem..

[23] Neumann U., Derwenskus F., Flister V.F., Schmid-Staiger U., Hirth T., Bischoff S.C. (2019). Fucoxanthin, a carotenoid derived from *Phaeodactylum tricornutum* exerts antiproliferative and antioxidant activities in vitro. Antioxidants.

[24] Zhang L., Wang H., Fan Y., Gao Y., Li X., Hu Z., Ding K., Wang Y., Wang X. (2017). Fucoxanthin provides neuroprotection in models of traumatic brain injury via the Nrf2-ARE and Nrf2-autophagy pathways. Sci. Rep..

[25] Rajauria G., Foley B., Abu-Ghannam N. (2017). Characterization of dietary fucoxanthin from *Himanthalia elongata* brown seaweed. Food Res. Int..

[26] Lourenço-Lopes C., Fraga-Corral M., Jimenez-Lopez C., Carpena M., Pereira A.G., Garcia-Oliveira P., Prieto M.A., Simal-Gandara J. (2021). Biological action mechanisms of fucoxanthin extracted from algae for application in food and cosmetic industries. Trends Food Sci. Technol..

[27] (2010). European Parliament and of the Council Directive 2009/32/EC on the approximation of the laws of the Member States on extraction solvents used in the production of foodstuffs and food ingredients. Off. J. Eur. Union.

[28] López C.J., Caleja C., Prieto M.A., Barreiro M.F., Barros L., Ferreira I.C.F.R. (2018). Optimization and comparison of heat and ultrasound assisted extraction techniques to obtain anthocyanin compounds from *Arbutus unedo* L. Fruits. Food Chem..

[29] Kemmer G., Keller S. (2010). Nonlinear least-squares data fitting in Excel spreadsheets. Nat. Protoc..

[30] Murado M.A., Prieto M.A. (2013). Dose-Response Analysis in the Joint Action of Two Effectors. A New Approach to Simulation, Identification and Modelling of Some Basic Interactions. PLoS ONE.

[31] Prikler S., de Levie R. (2009). Advanced Excel for Scientific Data Analysis.

[32] Heleno S.A., Diz P., Prieto M.A., Barros L., Rodrigues A., Barreiro M.F., Ferreira I.C.F.R. (2016). Optimization of ultrasound-assisted extraction to obtain mycosterols from *Agaricus bisporus* L. by response surface methodology and comparison with conventional Soxhlet extraction. Food Chem..

[33] Comuzzi C., Polese P., Melchior A., Portanova R., Tolazzi M. (2003). SOLVERSTAT: A new utility for multipurpose analysis. An application to the investigation of dioxygenated Co (II) complex formation in dimethylsulfoxide solution. Talanta.

[34] Lopes C.L., Pereira E., Soković M., Carvalho A.M., Barata A.M., Lopes V., Rocha F., Calhelha R.C., Barros L., Ferreira I.C.F.R. (2018). Phenolic Composition and Bioactivity of *Lavandula pedunculata* (Mill.) Cav. Samples from Different Geographical Origin. Molecules.

[35] Re R., Pellegrini N., Proteggente A., Pannala A., Yang M., Rice-Evans C. (1999). Antioxidant activity applying an improved ABTS radical cation decolorization assay. Free Radic. Biol. Med..

[36] Viacava G.E., Goyeneche R., Goñi M.G., Roura S.I., Agüero M.V. (2018). Natural elicitors as preharvest treatments to improve postharvest quality of Butterhead lettuce. Sci. Hortic..

[37] Bors W., Michel C., Saran M. (1984). Inhibition of the bleaching of the carotenoid crocin a rapid test for quantifying antioxidant activity. Biochim. Biophys. Acta (BBA)/Lipids Lipid Metab..

[38] Sokovic M., Glamoclija J., Marin M.D., Brkic D., van Griensven L.J.L.D. (2010). Antibacterial effects of the essential oils of commonly consumed medicinal herbs using an in vitro model. Molecules.

[39] Clinical and Laboratory Standards Institute (2012). Performance Standards for Antimicrobial Disk Susceptibility Tests: Approved Standard.

[40] Paz M., Gúllon P., Barroso M.F., Carvalho A.P., Domingues V.F., Gomes A.M., Becker H., Longhinotti E., Delerue-Matos C. (2015). Brazilian fruit pulps as functional foods and additives: Evaluation of bioactive compounds. Food Chem..

[41] Silva A., Silva S.A., Lourenço-Lopes C., Jimenez-Lopez C., Carpena M., Gullón P., Fraga-Corral M., Domingues V.F., Fátima Barroso M., Simal-Gandara J. (2020). Antibacterial use of macroalgae compounds against foodborne pathogens. Antibiotics.

[42] Lynne S.G. (2010). Clinical Microbiology Procedures Handbook.

[43] Ellman G.L., Courtney K.D., Andres V., Featherstone R.M. (1961). A new and rapid colorimetric determination of acetylcholinesterase activity. Biochem. Pharmacol..

[44] Ingkaninan K., Temkitthawon P., Chuenchom K., Yuyaem T., Thongnoi W. (2003). Screening for acetylcholinesterase inhibitory activity in plants used in Thai traditional rejuvenating and neurotonic remedies. J. Ethnopharmacol..

[45] Trott O., Olson A.J. (2009). AutoDock Vina: Improving the speed and accuracy of docking with a new scoring function, efficient optimization, and multithreading. J. Comput. Chem..

[46] Daina A., Michielin O., Zoete V. (2017). SwissADME: A free web tool to evaluate pharmacokinetics, drug-likeness and medicinal chemistry friendliness of small molecules. Sci. Rep..

[47] Zhang Y., Fang H., Xie Q., Sun J., Liu R., Hong Z., Yi R., Wu H. (2014). Comparative evaluation of the radical-scavenging activities of fucoxanthin and its stereoisomers. Molecules.

[48] Miyashita K., Beppu F., Hosokawa M., Liu X., Wang S. (2020). Bioactive significance of fucoxanthin and its effective extraction. Biocatal. Agric. Biotechnol..

[49] Guvatova Z., Dalina A., Marusich E., Pudova E., Snezhkina A., Krasnov G., Kudryavtseva A., Leonov S., Moskalev A. (2020). Protective effects of carotenoid fucoxanthin in fibroblasts cellular senescence. Mech. Ageing Dev..

[50] Ingkaninan K., De Best C.M., Van Der Heijden R., Hofte A.J.P., Karabatak B., Irth H., Tjaden U.R., Van Der Greef J., Verpoorte R. (2000). High-performance liquid chromatography with on-line coupled UV, mass spectrometric and biochemical detection for identification of acetylcholinesterase inhibitors from natural products. J. Chromatogr. A.

[51] Delerue T., Fátima Barroso M., Dias-Teixeira M., Figueiredo-González M., Delerue-Matos C., Grosso C. (2021). Interactions between *Ginkgo biloba* L. and *Scutellaria baicalensis* Georgi in multicomponent mixtures towards cholinesterase inhibition and ROS scavenging. Food Res. Int..

[52] Hu L., Chen W., Tian F., Yuan C., Wang H., Yue H. (2018). Neuroprotective role of fucoxanthin against cerebral ischemic/reperfusion injury through activation of Nrf2/HO-1 signaling. Biomed. Pharmacother..

[53] Sivagnanam S.P., Yin S., Choi J.H., Park Y.B., Woo H.C., Chun B.S. (2015). Biological properties of fucoxanthin in oil recovered from two brown seaweeds using supercritical CO_2_ extraction. Mar. Drugs.

[54] Karpiński T.M., Adamczak A. (2019). Fucoxanthin—An antibacterial carotenoid. Antioxidants.

[55] Rock C.O., Cronan J.E. (1996). *Escherichia coli* as a model for the regulation of dissociable (type II) fatty acid biosynthesis. Biochim. Biophys. Acta-Lipids Lipid Metab..

[56] Price A.C., Choi K.H., Heath R.J., Li Z., White S.W., Rock C.O. (2001). Inhibition of β-ketoacyl-acyl carrier protein synthases by thiolactomycin and cerulenin: Structure and mechanism. J. Biol. Chem..

[57] Sherer B.A., Hull K., Green O., Basarab G., Hauck S., Hill P., Loch J.T., Mullen G., Bist S., Bryant J. (2011). Pyrrolamide DNA gyrase inhibitors: Optimization of antibacterial activity and efficacy. Bioorganic Med. Chem. Lett..

[58] Maxwell A. (1997). DNA gyrase as a drug target. Trends Microbiol..

[59] Li X., Hilgers M., Cunningham M., Chen Z., Trzoss M., Zhang J., Kohnen L., Lam T., Creighton C., Gc K. (2011). Structure-based design of new DHFR-based antibacterial agents: 7-aryl-2,4-diaminoquinazolines. Bioorganic Med. Chem. Lett..

[60] Andrieu S., Coley N., Lovestone S., Aisen P.S., Vellas B. (2015). Prevention of sporadic Alzheimer’s disease: Lessons learned from clinical trials and future directions. Lancet Neurol..

[61] Anand P., Singh B., Singh N. (2012). A review on coumarins as acetylcholinesterase inhibitors for Alzheimer’s disease. Bioorganic Med. Chem..

[62] Dos Santos T.C., Gomes T.M., Pinto B.A.S., Camara A.L., De Andrade Paes A.M. (2018). Naturally occurring acetylcholinesterase inhibitors and their potential use for Alzheimer’s disease therapy. Front. Pharmacol..

[63] Li Q., Yang H., Chen Y., Sun H. (2017). Recent progress in the identification of selective butyrylcholinesterase inhibitors for Alzheimer’s disease. Eur. J. Med. Chem..

[64] Knez D., Coquelle N., Pišlar A., Žakelj S., Jukič M., Sova M., Mravljak J., Nachon F., Brazzolotto X., Kos J. (2018). Multi-target-directed ligands for treating Alzheimer’s disease: Butyrylcholinesterase inhibitors displaying antioxidant and neuroprotective activities. Eur. J. Med. Chem..

[65] Laskowski R.A., Furnham N., Thornton J.M. (2012). The Ramachandran plot and protein structure validation. Biomolecular Forms and Functions: A Celebration of 50 Years of the Ramachandran Map.

[66] Wróbel A., Arciszewska K., Maliszewski D., Drozdowska D. (2020). Trimethoprim and other nonclassical antifolates an excellent template for searching modifications of dihydrofolate reductase enzyme inhibitors. J. Antibiot..

[67] de Souza F.R., Guimarães A.P., Cuya T., de Freitas M.P., da Gonçalves A.S., Forgione P., Costa França T.C. (2017). Analysis of Coxiela burnetti dihydrofolate reductase via *in silico* docking with inhibitors and molecular dynamics simulation. J. Biomol. Struct. Dyn..

[68] Xiao H., Zhao J., Fang C., Cao Q., Xing M., Li X., Hou J., Ji A., Song S. (2020). Advances in Studies on the Pharmacological Activities of Fucoxanthin. Mar. Drugs.

[69] Waqar M., Batool S. (2015). *In silico* analysis of binding of neurotoxic venom ligands with acetylcholinesterase for therapeutic use in treatment of Alzheimer’s disease. J. Theor. Biol..

[70] Shiri F., Pirhadi S., Ghasemi J.B. (2019). Dynamic structure based pharmacophore modeling of the Acetylcholinesterase reveals several potential inhibitors. J. Biomol. Struct. Dyn..

[71] Xiang S., Liu F., Lin J., Chen H., Huang C., Chen L., Zhou Y., Ye L., Zhang K., Jin J. (2017). Fucoxanthin Inhibits β-Amyloid Assembly and Attenuates β-Amyloid Oligomer-Induced Cognitive Impairments. J. Agric. Food Chem..

[72] Alghazwi M., Smid S., Musgrave I., Zhang W. (2019). In vitro studies of the neuroprotective activities of astaxanthin and fucoxanthin against amyloid beta (Aβ 1-42) toxicity and aggregation. Neurochem. Int..

[73] Lin J., Huang L., Yu J., Xiang S., Wang J., Zhang J., Yan X., Cui W., He S., Wang Q. (2016). Fucoxanthin, a marine carotenoid, reverses scopolamine-induced cognitive impairments in mice and inhibits acetylcholinesterase in vitro. Mar. Drugs.

[74] Daina A., Zoete V. (2016). A BOILED-Egg To Predict Gastrointestinal Absorption and Brain Penetration of Small Molecules. ChemMedChem.

[75] Paudel P., Seong S.H., Jung H.A., Choi J.S. (2019). Characterizing fucoxanthin as a selective dopamine D3/D4 receptor agonist: Relevance to Parkinson’s disease. Chem. Biol. Interact..

[76] Hashimoto T., Ozaki Y., Taminato M., Das S.K., Mizuno M., Yoshimura K., Maoka T., Kanazawa K. (2009). The distribution and accumulation of fucoxanthin and its metabolites after oral administration in mice. Br. J. Nutr..

